# Job tenure and work injuries: a multivariate analysis of the relation with previous experience and differences by age

**DOI:** 10.1186/1471-2458-13-869

**Published:** 2013-09-22

**Authors:** Antonella Bena, Massimiliano Giraudo, Roberto Leombruni, Giuseppe Costa

**Affiliations:** 1 Department of Epidemiology, Servizio di Epidemiologia – Settore rischi e danni da lavoro – ASL TO3 – Grugliasco, Via Sabaudia 164 Grugliasco, Turin 10095, Italy; 2 Department of Economics "Cognetti de Martiis", University of Turin, Turin, Italy; 3 Department of Clinical and Biological Sciences, University of Turin, Orbassano, Turin, Italy

**Keywords:** Occupational injuries, Epidemiology, Longitudinal studies, Age factors, Time factors, Flexibility, Work experience, Job tenure, Career mobility

## Abstract

**Background:**

One of the consequences of the increasing flexibility in contemporary labour markets is that individuals change jobs more frequently than in the past. Indeed, in many cases, through collecting a lot of contracts, individuals work in the same economic sector or even in the same company, doing the same job in the same way as existing colleagues. A very long literature has established that newly hired workers – whatever the contract type – are more likely to be injured than those with longer job tenures. The objectives of this paper are: 1) to study the relationship between job tenure and injury risk taking into account past experience as a possible confounder; and 2) to evaluate how the effects of past experience and job tenure are modified by age.

**Methods:**

Using a longitudinal national database, we considered only job contracts starting in 1998–2003 held by men working as blue collars or apprentices in the non-agricultural private sector. We calculated injury rates stratified by job tenure and age. Multivariate analyses were adjusted for background variables and previous experience accrued in the same economic sector of the current job.

**Results:**

In the study period 58,271 workers who had experienced 10,260 injuries were observed. These people worked on 115,277 contracts in the six years observed (1.98 contracts per worker). Injury rates decrease with job tenure; the trend is the same in each age group; young workers have both the highest injury rate (9.20; CI 95%: 8.95-9.45) and the highest decrease with job tenure. Previous experience is associated with a decreasing injury rate in all age groups and for all job tenures. Multivariate analyses show that, even after checking for previous experience, workers with job tenure of less than 6 months show always higher relative risks compared with job tenure > 2 years: relative risk is 41% higher among under-thirty workers; it is 22% higher among people over forty. Previous experience is protective against injury risk in workers over thirty: after checking for all other variables, relative risk is lower in workers who have accumulated more than 5 years of experience.

**Conclusions:**

In a context in which career fragmentation is increasing, workers find themselves more and more in the "high risk" period and only individuals who are able to build their career with similar jobs may mitigate the higher risks thanks to their past experience. If institutions don’t adopt appropriate prevention policies, injury risk is likely to increase, especially among young people.

## Background

Temporary employment is increasing throughout the world. In Italy, the share of temporary contracts among workers between 15 and 64 years of age rose from 9.8% in 1999 to 13.4% in 2011.

In 2011, half of workers between 15 and 24 years of age had a temporary contract. The percentages are comparable with other European countries [1].

Most published research documents the adverse effects of temporary work on health, although null findings have also been reported [2]. A recent review suggests that temporary employment might be associated with lower sickness absence rates than permanent employment [3], even if some studies did not find this association after controlling for individual situations and job characteristics [4,5]. In the case of work injuries, the review concluded that only seven out of thirteen studies have shown higher risk for fixed-term workers compared to permanent ones.

Among the factors that have been identified as explaining the association between temporary employment and work injuries are the assignment of temporary workers to occupations with more hazardous working conditions and a lack of safety training in workplaces [6-8]. Another common explanation states that the higher injury incidence is a result of less work experience. This second explanation suggests a more general link between the increasing flexibility of contemporary labour markets, where individuals change jobs more frequently than in the past, and workplace safety. Indeed, a very long literature has established that newly hired workers – whatever the contract type – are more likely to get injured than those with longer job tenures [9,10]. The increased flexibility however is a contextual factor that modified the way in which this relation operates:

1. On the one hand, the increased flexibility often results in the fragmentation of the entire career. Short-term job tenure is no longer mainly associated with young people entering the labour force [11]: individuals now frequently collect many contracts during their entire working life and this may put them continuously in the “high risk” period of starting a job.

2. On the other hand, a worker starting a new contract after having already had many jobs should not be considered *tout court (wholly)* without experience.

Most studies have focussed mainly on investigating whether the short job tenures of temporary employment are associated with higher injury risks, without considering the potential protective effect of past experience. Yet, in many cases, through collecting a lot of contracts, individuals work in the same economic sector or even in the same company, doing the same job in the same way as colleagues with permanent contracts. This may confound the relation between job tenure and injuries, particularly at different ages and may lead to an overestimation of the public health concerns about temporary employment if career experience proves to be a substitute for within-the-job experience.

The aim of this paper is to study whether the experience accumulated prior to the beginning of a contract may have a protective role against work injuries. In particular the objectives are: 1) to study the relationship between time on the job (that is *job tenure*) and injury risk, taking into account past experience as a possible confounder, and 2) to evaluate how the effects of past experience and job tenure are modified by age.

## Methods

### The WHIP-INAIL data base

The study is based on the WHIP-INAIL archive, described in detail elsewhere [12].

In short, the Work History Italian Panel (WHIP) is the result of data processing of a 1% sample of individuals taken from the archives of the Italian National Social Security Institute (INPS). INPS insures approximately 15 million people: employees in private companies, semi-dependent workers and self-employed people (artisans and traders) with the exclusion of professionals like lawyers or architects. This group effectively represents all activities in manufacturing, construction and services in Italy; while public employment, predominant in education and health sectors, is not covered. Moreover there are other sectors, such as agriculture, which are not entirely comprised in INPS. For each person sampled the employment history was reconstructed, including all employment spells, retirement, and any period in which the individual received social security benefits such as unemployment subsidy. The period covered is from 1985 to 2004. The data regarding dependent employment (used in this paper) include basic demographic information (age, sex, country of birth), information about the job (such as weeks worked, skill level, type of contract, temporary leaves, dates of job start and end), plus basic data about the employer (date of constitution, economic sector, yearly average number of employees).

The Italian Workers Compensation Authority (INAIL) collects information on occupational injuries with an absence from work of more than three days certified by a physician (always required). The injuries occurred between 1994 and 2003 were extracted from the national database with the same sampling frame used for the WHIP archive. Injuries were deterministically linked with the WHIP sample using an encrypted version of the Italian tax code, which is issued by the Italian tax office to unambiguously identify all individuals residing in Italy. All activities, regardless of their complexity or depth, were conducted in accordance with Italian regulations on privacy and with the approval of the national institutes involved. The accuracy of the WHIP-INAIL estimate of the injury risk was demonstrated by a comparison with Eurostat statistics and by the consistency of preliminary results with published data [12].

### Statistical analysis

In the analysis we considered only job contracts in private companies starting in the period 1998–2003, held by men working as blue collars or apprentices in the non-agricultural private sector. We chose to focus the analysis in the last period available to avoid any bias related to changes occurring in the labor market regulation over the years (in 1997 there was an important reform in the regulation of the labor market – law num. 196/1997). However, for the workers selected in the study, we use all the information in the database, considering their career also prior to 1998.

In total we selected 115,277 contracts (representing 74% of contracts in the data set), both permanent and temporary. The latter included fixed-term contracts, seasonal work and on-the-job training contracts, while jobs obtained through temporary work agencies were excluded, since for these it is impossible to identify important confounders such as economic sector and firm size at the time of the injury as they are recorded in the INPS archives under the name of the agencies rather than the employer.

We distinguished two types of experience: the first is the one accrued within the job contract currently held by a worker, which we call the *job tenure*; the second is the one accrued before the current job contract, which we call the *previous experience*.

The job tenure is a time dependent variable calculated as the time elapsed from the beginning of the contract. Its length is maximum 72 months. It was categorized into 4 classes (< 6 months; 6–12 months; 13–24 months; >24 months).

For example, a worker who begins to work on 01/01/2003 and ends to work on 31/12/2003 was classified:

•in the class <6 months of job tenure for the period 01/01/2003-31/05/2003

•in class 6–12 months of job tenure for the period 01/06/2003-31/12/2003.

A worker who begins to work on 01/01/1999 and ends to work on 31/12/2003 was classified:

in the class <6 months for the period 01/01/1999-31/05/1999;

in the class 6–12 months for the period 01/06/1999-31/12/1999;

in the class 13–24 months for the period 01/01/2000-31/12/2000;

in the class >24 months for the period 01/01/2001-31/12/2003.

To classify workers according to their *previous experience*, workers never registered in the data base prior to their current job were defined as “workers with no previous experience”. For all other workers the previous experience is calculated as the number of years spent as an employee or self-employed in the same economic sector of the current job. It was categorized into 3 classes (<1 year; 1–5 years; >5 years).

*Time at risk* was calculated on the basis of months actually worked, which were obtained subtracting from paid months all periods of absence from work due to illness or injuries, temporary-lay-off and maternity. All analyses were stratified by age class (< 30 years; 31–40 years; > 40 years).

In our analysis we considered only injuries occurred in 1998–2003. Injury rates per 100 person years were calculated (with the relative confidence intervals at 95%) for job tenure and previous experience stratified by age.

Injury risks for job tenure, stratified by age, were calculated using a Poisson distribution for panel data that takes into account time-dependent variables. The 95% confidence intervals were calculated applying the correction for repeated events [13]. We tested three models:

model 1: unadjusted;

model 2: adjusted for background variables: country of birth, year of birth, economic sector (11 classes, among C-I codes of the statistical classification of economic activities in the European Community NACE), firm size (yearly average number of employees), firm geographic area (based on Italy’s administrative boundaries);

model 3: model 2 plus previous experience.

Finally, we calculated injury risks for previous experience stratified by age and adjusted for background variables and job tenure.

Analyses were performed using SAS 9.2 and Stata 10.

## Results

In the study period 58,271 workers who had experienced 10,260 injuries were observed. These people worked on 115,277 contracts in the six years observed (1.98 contracts per worker). Table 1 shows the main characteristics of population included in the study. As expected, among workers who began a new contract (Table 2), there were mostly people under 30 years of age (47% of person years); however, the phenomenon was relevant also for workers over forty (23% of person years). Young people changed contract more frequently: among workers with a job tenure shorter than 6 months, 52% of person years were under thirty; those with a duration > 24 months accounted for 40%. Young people had less previous experience: among the workers with no previous experience, 75% of person years was under 30. On the contrary 48% of person years with previous specific experience longer than 5 years were over 40.

**Table 1:** Injuries and person years stratified per age, country of birth, economic sector, firm size, firm geographic area

		**Age**									**Total**		
		**< 30**			**31-40**			**> 40**					
		**Num. of injuries**	**Person years**		**Num. of injuries**	**Person years**		**Num. of injuries**	**Person years**		**Num. of injuries**	**Person years**	
			**N**	**%**		**N**	**%**		**N**	**%**		**N**	**%**
**Country of birth**													
	Italy	4410	48089	48.60	2142	26974	27.26	1730	23887	24.14	8282	98950	100
	Others	783	8347	41.03	858	8429	41.44	337	3565	17.53	1978	20341	100
**Economic sector**													
	Mining and quarrying	27	194	31.89	18	197	32.44	20	217	35.67	65	609	100
	Manufacture of food products. beverages and tobacco	172	2038	47.02	99	1302	30.05	69	993	22.92	340	4333	100
	Manufacture of textiles and textile products	102	1442	47.27	52	1001	32.81	30	608	19.93	184	3050	100
	Manufacture of chemicals. chemical products. man-made fibres. rubber and plastic products	233	2223	49.94	129	1402	31.50	66	826	18.56	428	4451	100
	Manufacture of basic metals. fabricated metal products. electrical. optical and transport equipment	1758	15718	53.68	896	8282	28.28	489	5281	18.04	3143	29281	100
	Other manufacture	592	6607	47.42	365	4286	30.77	202	3038	21.81	1159	13931	100
	Electricity. gas and water supply	7	104	27.96	16	128	34.33	9	140	37.71	32	372	100
	Construction	1146	12161	41.48	743	8367	28.54	755	8793	29.99	2644	29320	100
	Wholesale and retail trade; repair of motor vehicles. motorcycles and personal and household goods	531	8131	52.70	263	4382	28.40	161	2916	18.90	955	15429	100
	Hotels and restaurants	247	4184	50.17	97	2369	28.41	50	1787	21.42	394	8339	100
	Transport. storage and communication	378	3635	35.73	322	3688	36.25	216	2851	28.03	916	10174	100
**Firm size (yearly average number of employees)**													
	0-9	1290	18197	51.45	617	9683	27.38	465	7488	21.17	2372	35369	100
	10-19	746	7354	46.69	444	4760	30.22	269	3637	23.09	1459	15752	100
	20-49	716	6575	45.66	457	4398	30.54	281	3426	23.79	1454	14399	100
	50-99	414	3453	44.75	275	2415	31.30	184	1848	23.95	873	7716	100
	> 99	1023	7804	50.15	516	4507	28.96	322	3251	20.89	1861	15562	100
	Missing	1004	13051	42.80	691	9639	31.61	546	7802	25.59	2241	30492	100
**Firm geographic area**													
	Northwest	1633	17289	49.93	924	10216	29.50	572	7124	20.57	3129	34629	100
	Northest	1683	15021	50.08	953	8855	29.52	576	6116	20.39	3212	29992	100
	Central	886	9997	46.96	562	6418	30.15	356	4873	22.89	1804	21288	100
	South and Islands	991	14129	42.33	561	9914	29.70	563	9338	27.97	2115	33381	100
**Total**		5193	56436	47.3	3000	35403	29.68	2067	27452	23.01	10260	119290	100

**Table 2:** Injuries and person years stratified per age, job tenure and previous experience

		**Age**									**Total**		
		**< 30**			**31-40**			**> 40**					
		**Num. of injuries**	**Person years**		**Num. of injuries**	**Person years**		**Num. of injuries**	**Person years**		**Num. of injuries**	**Person years**	
			**N %**			**N %**			**N %**			**N %**	
Job Tenure													
	< 6 months	1883	17051	52.21	869	8775	26.87	572	6832	20.92	3324	32658	100
	6-12 months	1334	14093	49.76	735	8066	28.48	481	6161	21.75	2550	28320	100
	13-24 months	1083	13272	46.63	688	8646	30.38	479	6545	22.99	2250	28463	100
	> 24 months	893	12020	40.27	708	9916	33.22	535	7914	26.51	2136	29850	100
Previous Experience													
Workers with no previous experience		1158	15716	74.95	250	3581	17.08	89	1672	7.97	1497	20969	100
Workers with previous experience	< 1 year	2749	25970	53.33	1357	14173	29.11	741	8550	17.56	4847	48693	100
	1 - 5 years	1011	11351	47.32	699	7807	32.55	403	4828	20.13	2113	23986	100
	> 5 years	275	3398	13.25	694	9841	38.38	834	12401	48.37	1803	25640	100
Total		5193	56436	47.31	3000	35403	29.68	2067	27452	23.01	10260	119290	100

Table 3 shows injury rates stratified by job tenure, age and previous experience. Injury rates decrease with increase in job tenure (< 6 months: 10.20; CI 95%: 9.83-10.52; > 2 years: 7.16; CI 95%: 6.85-7.46); the trend is the same in each age group considered. Workers under 30 years of age have the highest injury rate (9.20; CI 95%: 8.95-9.45), statistically higher if compared with the other age groups (31–40 years: 8.47; CI 95%: 8.17-8.78; > 40 years: 7.53; CI 95%: 7.20-7.85). Previous experience is associated with a decreasing injury rate in all age groups and for all job tenures. Workers with no previous experience have the lowest injury rates in all ages and all job tenures considered.

**Table 3:** Injury rates per 100 workers by job tenure and previous experience, stratified by age

**Job tenure**	**Previous experience**		**<= 30**		**31-40**		**> 40**		**Total**	
			**Rate**	**CI 95%**	**Rate**	**CI 95%**	**Rate**	**CI 95%**	**Rate**	**CI 95%**
< 6 months										
	Workers with no previous experience		8.95	(8.09-9.82)	5.88	(4.31-7.45)	3.67	(1.81-5.53)	8.11	(7.38-8.84)
	Workers with previous experience	< 1 year	12.9	(12.11-13.69)	12.1	(10.96-13.32)	10.9	(9.42-12.32)	12.4	(11.81-13.01)
		1-5 years	10.17	(9.11-11.23)	10.8	(9.36-12.15)	8.91	(7.32-10.49)	10.1	(9.35-10.85)
		> 5 years	8.92	(7.07-10.76)	7.53	(6.43-8.64)	7.16	(6.22-8.10)	7.57	(6.90-8.24)
	Total		11.04	(10.54-11.54)	9.9	(9.24-10.56)	8.37	(7.69-9.06)	10.2	(9.83-10.52)
6-12 months										
	Workers with no previous experience		7.43	(6.58-8.29)	7.96	(6.13-9.79)	3.8	(1.94-5.66)	7.23	(6.51-7.96)
	Workers with previous experience	< 1 year	10.83	(10.02-11.63)	10.3	(9.11-11.38)	8	(6.70-9.30)	10.2	(9.63-10.80)
		1-5 years	9.76	(8.61-10.90)	9.76	(8.33-11.19)	9.01	(7.23-10.80)	9.62	(8.81-10.42)
		> 5 years	7.75	(5.95-9.56)	7.52	(6.39-8.65)	7.82	(6.79-8.85)	7.7	(7.00-8.40)
	Total		9.47	(8.96-9.97)	9.11	(8.45-9.77)	7.81	(7.11-8.50)	9	(8.65-9.35)
13-24 months										
	Workers with no previous experience		6.65	(5.82-7.48)	6.73	(4.95-8.51)	9.66	(6.50-12.82)	6.89	(6.16-7.63)
	Workers with previous experience	< 1 year	9.14	(8.38-9.90)	8.8	(7.82-9.79)	8.06	(6.84-9.28)	8.85	(8.31-9.39)
		1-5 years	7.93	(6.86-9.01)	8.08	(6.79-9.36)	8.47	(6.74-10.20)	8.08	(7.34-8.83)
		> 5 years	8.46	(6.48-10.44)	7.09	(6.04-8.14)	6.1	(5.22-6.99)	6.8	(6.16-7.44)
	Total		8.16	(7.67-8.65)	7.96	(7.36-8.55)	7.32	(6.66-7.97)	7.91	(7.58-8.23)
> 2 years										
	Workers with no previous experience		5.97	(5.16-6.79)	7.32	(5.58-9.06)	4.69	(2.73-6.65)	6.11	(5.41-6.80)
	Workers with previous experience	< 1 year	8.55	(7.78-9.32)	7.71	(6.88-8.54)	7.96	(6.89-9.03)	8.13	(7.63-8.63)
		1-5 years	7.14	(6.07-8.21)	7.1	(5.92-8.27)	7.1	(5.65-8.55)	7.12	(6.42-7.81)
		> 5 years	6.82	(4.80-8.83)	6.21	(5.27-7.14)	5.98	(5.17-6.80)	6.15	(5.56-6.74)
	Total		7.43	(6.94-7.92)	7.14	(6.61-7.67)	6.76	(6.19-7.33)	7.16	(6.85-7.46)
Total										
	Workers with no previous experience		7.37	(6.94-7.79)	6.98	(6.12-7.85)	5.32	(4.22-6.43)	7.14	(6.78-7.50)
	Workers with previous experience	< 1 year	10.59	(10.19-10.98)	9.57	(9.06-10.08)	8.67	(8.04-9.29)	9.95	(9.67-10.23)
		1-5 years	8.91	(8.36-9.46)	8.95	(8.29-9.62)	8.35	(7.53-9.16)	8.81	(8.43-9.18)
		> 5 years	8.09	(7.14-9.05)	7.05	(6.53-7.58)	6.73	(6.27-7.18)	7.03	(6.71-7.36)
	Total		9.2	(8.95-9.45)	8.47	(8.17-8.78)	7.53	(7.20-7.85)	8.6	(8.43-8.77)

Table 4 shows the results of the multivariate analysis stratified by age: compared with job tenure > 2 years, workers with job tenure of less than 6 months show always higher relative risks (first model): relative risk is 46% (CI 95%: 1.33-1.59) higher among workers under 30 years of age; it is 21% (CI 95% 1.07-1.73) higher among people over forty. The relative risks do not change much after controlling for background variables (second model). The most important confounding factors are firm size and economic sector (data not shown). The relative risks do not change even after controlling for previous experience (third model).

**Table 4:** Injury rates by job tenure stratified by age and controlled for background variables and previous experience

**Job tenure**		**Age**					
		**<= 30**		**31-40**		**> 40**	
		**RR**	**CI 95%**	**RR**	**CI 95%**	**RR**	**CI 95%**
Model 1: unadjusted							
	< 6 months	1.46	(1.33 – 1.59)	1.35	(1.22 – 1.50)	1.21	(1.07 – 1.37)
	6-12 months	1.27	(1.16 – 1.39)	1.26	(1.13 – 1.40)	1.14	(1.01 – 1.30)
	13-24 months	1.08	(0.99 – 1.19)	1.11	(1.00 – 1.23)	1.08	(0.95 – 1.22)
	> 24 months	1.00		1.00		1.00	
Model 2: adjusted for economic sector, firm size, country of birth, firm geographic area, year of birth							
	< 6 months	1.43	(1.31 – 1.57)	1.32	(1.19 – 1.47)	1.22	(1.08 – 1.38)
	6-12 months	1.23	(1.13 – 1.35)	1.21	(1.09 – 1.35)	1.12	(0.99 – 1.28)
	13-24 months	1.05	(0.96 – 1.16)	1.07	(0.97 – 1.19)	1.06	(0.94 – 1.20)
	> 24 months	1.00		1.00		1.00	
Model 3: model 2 plus previous experience							
	< 6 months	1.41	(1.29 – 1.55)	1.32	(1.19 – 1.47)	1.22	(1.08 – 1.39)
	6-12 months	1.22	(1.11 – 1.34)	1.22	(1.09 – 1.36)	1.13	(0.99 – 1.28)
	13-24 months	1.04	(0.95 – 1.15)	1.07	(0.97 – 1.19)	1.06	(0.94 – 1.20)
	> 24 months	1.00		1.00		1.00	

Previous experience is protective against injury risk in workers over thirty (Table 5): after controlling for all other variables, RR is lower in workers with more than 5 years experience. Workers with no previous experience have a lower RR in all age groups, but only among workers over forty this becomes statistically significant (RR: 0.68; CI 95%: 0.53-0.88).

**Table 5:** Injury rates by previous experience stratified by age and controlled for background variables and job tenure

**Previous experience**		**Age**					
		**<= 30**		**31-40**		**> 40**	
		**RR**	**CI 95%**	**RR**	**CI 95%**	**RR**	**CI 95%**
Model 1: unadjusted							
Workers with no previous experience		0.87	(0.73 - 1.05)	1.00	(0.85 - 1.16)	0.80	(0.63 - 1.01)
Workers with previous experience	< 1 year	1.23	(1.03 - 1.46)	1.36	(1.23 - 1.50)	1.29	(1.16 - 1.44)
	1 – 5 years	1.04	(0.87 - 1.25)	1.27	(1.13 - 1.42)	1.25	(1.09 - 1.42)
	> 5 years	1.00		1.00			
Model 2: adjusted for job tenure, economic sector, firm size, country of birth, firm geographic area, year of birth							
Workers with no previous experience		0.85	(0.70 – 1.03)	0.88	(0.75 – 1.05)	0.68	(0.53 – 0.88)
Workers with previous experience	< 1 year	1.09	(0.91 – 1.30)	1.21	(1.09 – 1.34)	1.21	(1.08 – 1.36)
	1 – 5 years	1.01	(0.84 – 1.21)	1.24	(1.10 – 1.39)	1.22	(1.07 – 1.39)
	> 5 years	1.00		1.00		1.00	

## Discussion

Our findings show an inverse relationship between job tenure and injury risk: results estimated on the Italian cohort are consistent with studies conducted in other countries [7,9,10]. This relationship concerns all ages, but the risk differences are more evident among people under 30 years of age. The relationship persists despite the effect of confounding due to economic sector, firm size, country of birth and firm geographic area (model 2). This work is the first of this kind in Italy: the studies available up to the time of our research were limited to workers hired from temporary work agencies and did not take into account confounding factors [14-16].

The present study makes some important contributions to understanding the relationship between work injuries and careers characterized by many work contracts of short duration. The two factors that shape this relation are job tenure and past experience: to our knowledge, there have been no published papers assessing the role of the latter factor in determining injuries. Usually studies assessing the influence of experience on safety take into consideration only on-the-job experience (that is *job tenure*); Saloniemi emphasizes the importance of reconstructing the work histories of fixed-term workers to evaluate experience as a whole [17].

Firstly, previous experience has a protective role against injuries for workers over 30 years old (see Table 5). Compared to the colleagues with more than 5 years experience, workers with less than 5 years of experience have a 20% higher injury risk. Below 5 years of experience, there appear to be no differences among workers with less than 1 year or between 2 to 5 years of experience. This result seems to exclude a gradient-like shape of the effect, instead it points to a bipartition between workers with or without a long (five years and over) specific experience. This is coherent with the absence of an effect on workers under 30 years of age, who – due to their age – will rarely have a specific experience significantly higher than five years.

Our results show that increased mobility across jobs as well as amongst workers under 30 years of age may have different effects, depending on whether workers are able to re-employ themselves in jobs requiring their specific work experience.

Previous experience, however, is not a modifier of the relation between injury risk and job tenure (see Table 4): in all age classes, newly hired workers show a relative risk of injury which is the same with or without controlling for previous work experience (model 3). This result shows that previous experience is only a partial substitute of the experience accrued within the job. Although it is protective factor, it is unable to support workers in facing all the demands of a new organization: it would take at least one year of work in a new company to acquire the familiarity needed to work in a comparable way to colleagues employed the longest time. The increased injuries risk would be related to work organization and to interaction with colleagues [18]. Newly hired workers tend to perform unfamiliar work or tasks that were not part of their previous duties. In a previous study conducted in the USA, “using a different method to do a task” and “doing an unusual task” were found to significantly increase the relative risk of an occupational acute hand injury [19]. The newly hired are less familiar with the formal and informal rules governing safety on site and their presence may affect inter-worker communications. In other words, it becomes more difficult to coordinate decisions, to anticipate dangers and to recognize the consequences of particular individuals or groups controlling materials and processes. Another explanation for high injury risk in job tenures of less than 1 year may be due to the assignment of newly hired workers to high-risk activities that existing workers refuse to do [18]. To contrast such risks, the management of a company should outline an initial phase of *adaptation* during which new employees are properly introduced to the company’s organization with the support of colleagues who already have experience in the company and during which they are assigned to simpler and less dangerous tasks. A study based on a survey conducted on 300 Italian workers employed by temporary work agencies found similar results [14].

Workers under 30 years of age show a stronger job tenure/injury association than older workers. Our multivariate analysis shows that neither background characteristics nor previous experience change this distinction. There are no obvious mechanisms that would help explain this fact. Further research is needed to investigate whether in Italy there are differences by age in safety training at the beginning of a new job as reported in other countries [6]. Another important issue concerns the existence of age differences in risk perception that could put young workers in situations of greater danger compared to older colleagues [20]. The two most relevant reviews of age differences [21,22] indicate that a substantial part of the elevated injury risk experienced by young workers appears to be due to differences in the types of jobs held by young people and adults (and the associated hazard exposures). Although temporary work is a phenomenon that concerns the entire workforce, it is more common among young people and our results suggest it is important to include job tenure among the control variables when studying injury risk in young workers [23]. Further research is needed to focus on this age class, in particular on the fact that certain age groups may be more likely to have unfavourable working conditions.

All analyses show that injury rates are always lower among workers with no previous experience. We think that this is the result of two different selection processes.

Among workers under 30 years of age, those with no previous experience were mostly represented by young people entering the labour market for the first time: they had an average age of 21, a contract of apprenticeship in 40% of cases and an average wage of 873 euro per month. For all those entering the market, the best worker-firm matches on average will survive longer and individuals in these (presumably safer) jobs will remain classified as “workers with no previous experience”. On the other hand, workers with worse jobs and/or worse health are more likely to leave their first job and have another or many other contracts. This sort of “healthy worker survivor effect” will imply that on average the young who are no longer in their first job will probably be working in worse and more dangerous jobs.

Among people over thirty, workers with no previous experience are mostly immigrants (< 30 years: 28%; 31–40 years: 78%; > 40 years: 64%). In this case there is probably a selection mechanism of healthy workers with experience gained in foreign firms. In fact, some studies showed evidence of a “healthy immigrant effect” whereby immigrants, and especially recent immigrants, are less likely than indigenous population to have poorer health [24]. In regards to workers over 30 years of age born in Italy who enter in the INPS data-base for the first time (very few indeed in our sample, see the confidence intervals), they mostly belong to particular categories previously recorded in other public social security funds. So, for the large part these are fake entries: the typical situation is that of a worker employed in a large public company, which is then privatized. He /she, on average, will be working in a safer work environment.

Interpretation of our findings must be made in light of study limitations.

Our research was restricted to male workers in the private sector, employed as blue collars or apprentices, mainly in manufacturing, construction and services: this way we reduced the likelihood that other job characteristics and selection processes might bias our estimates [3]. The results for this workers' category are quite reliable but, in order to test whether our conclusions can be generalized to the entire Italian population, we are conducting further analyses on women, the self-employed and white-collars. Farm workers and public employees are insured by other social security funds. Since the Italian government has recently assigned the management of these funds to INPS, we hope in the future to be able to extend our analysis to these workers too.

This paper does not consider the type of injuries. The risks due to age, experience and job tenure may be different, for example, for fall and handling machines/objects. Further analyses devoted to specific injury types would be advisable.

Previous experience was calculated without distinguishing the length and number of contracts that had generated it. For example, a worker with a previous two-year contract has been equated to a person with four previous contracts of six months each. Furthermore, we didn't consider whether the experience was gained in the same company or in different companies and we didn’t take into account periods out of work, where the possibility of a depreciation of human capital should be considered. In order to assess in more detail if these differences in worker careers have a specific effect on injury risks, we are reconstructing the careers of every single worker in the WHIP-INAIL archive.

Jobs obtained through temporary work agencies are excluded because they are recorded in INPS archives under the agencies, so it’s impossible to identify the economic sector and firm size at the time of the injury. We believe that injury risks aren’t underestimated since they were not very numerous in the study period (in 2008 they represented 4.5% of workers with temporary contracts [25]; 1.6% of time at risk in our analyses). Although we could not include workers that obtained jobs through temporary work agencies in the study, our results raise great concern about these subjects: they work in the same firm only for very short periods, hence incurring the high risks that are linked to the short tenure. Moreover, since they are mostly of young age and may often change type of job, it is unlikely that they would have been able to acquired the long specific experience needed to countervail the effects of the short tenure.

## Conclusions

Our analysis shows that job tenure is inversely associated with injury risks even taking into account background variables and previous experience. People under 30 years of age show higher risk rates but the phenomenon does not spare other age groups; previous experience is protective against injury risk among workers over thirty but not among young people. These results, in a context in which labour market flexibility is increasing and job switches are more common, mean that workers find themselves more and more in the "high risk" period and only individuals who are able to build their career with similar jobs may mitigate the higher risks thanks to their past experience. If institutions do not adopt appropriate prevention policies, injury risk is likely to increase, especially among young people. It is also evident that it is important to adopt appropriate strategies at company level: management should outline safety systems in which new employees are properly introduced in the company’s organization with the support of colleagues who already work in the company and they should also be assigned to simpler and less dangerous tasks. The current economic crisis could modify these results. Research has shown an inverse relationship between unemployment rate and morbidity among temporary workers: in this situation, employers are more likely to find and recruit healthy workers (into temporary jobs) from the reserve of unemployed people [3]. Surveillance activities on injury risk will need to take into account the presence of contextual effects like this, which may hide medium term structural trends.

The causal effect of career fragmentation on injury and the relative contribution of several selection processes should be examined with prospective study designs, tracking the change from one employment status to another. The WHIP-INAIL data-base allows the tracking of careers and, in the near future, will provide data allowing the extension of the samples and the inclusion of new morbidity data. This will allow the study of other effects of labour market flexibility on health in Italy.

## Abbreviations

WHIP: Work Histories Italian Panel; INAIL: Italian workers compensation authority; INPS: Italian National Social Security Institute; CI: Confidence interval.

## Competing interests

The authors declare that they have no competing interest.

## Authors’ contributions

AB was the main supervisor of the project, and wrote the paper; MG performed all the statistical analyses; RL contributed to the conception of the work and commented on the analyses; GC read and commented on the paper. Each author is confident in the validity of this work, has reviewed the final version of the manuscript and approves it for submission.

## Pre-publication history

The pre-publication history for this paper can be accessed here:

http://www.biomedcentral.com/1471-2458/13/869/prepub
